# Molnupiravir Inhibits Porcine Epidemic Diarrhea Virus Infection In Vitro

**DOI:** 10.3390/v15061317

**Published:** 2023-06-02

**Authors:** Zi-Xin Huang, Shu-Ting Zhou, Zhi-Biao Yang, Zhe Wang

**Affiliations:** 1 Shanghai Collaborative Innovation Center of Agri-Seeds, School of Agriculture and Biology, Shanghai Jiao Tong University, Shanghai 200240, China; 2 Shanghai Key Laboratory of Veterinary Biotechnology, School of Agriculture and Biology, Shanghai Jiao Tong University, Shanghai 200240, China

**Keywords:** PEDV, molnupiravir, antiviral, RdRp activity, RNA-Seq

## Abstract

Porcine epidemic diarrhea virus (PEDV) is a swine coronavirus that is highly infectious and prone to variation. Vaccines derived from traditional PEDV strains provide less protection against PEDV-variant strains. Furthermore; there is a complex diversity of sequences among various PEDV-variant strains. Therefore; there is an urgent need to develop alternative antiviral strategies to defend against PEDV. Molnupiravir is a nucleotide analogue that could replace natural nucleosides to restrain viral RNA replication. Our study provided evidence for the dose-dependent inhibition of PEDV replication by molnupiravir in Vero cells. Molnupiravir also exhibited a strong inhibitory effect on viral RNA and protein production. Our results demonstrated that molnupiravir inhibits PEDV RNA-dependent RNA polymerase (RdRp) activity and induces a high frequency of mutations in the PEDV genome. Further studies revealed that molnupiravir can reverse changes in the transcriptome caused by viral infection. In conclusion, our results indicated that molnupiravir has the potential to be an effective treatment for PEDV infection.

## 1. Introduction

Porcine epidemic diarrhea (PED) is a disease characterized by emesis, watery diarrhea, and severe dehydration in pigs caused by porcine epidemic diarrhea virus (PEDV). It was first isolated in Belgium in the 1970s [1]. Currently, PEDV is primarily classified into two genogroups: the GI genogroup and the GII genogroup. The G1 genogroup represents classical strains, while the G2 genogroup represents variant strains. These two groups have been subdivided into five subgroups (GIa, GIb, GIIa, GIIb, and GIIc) [2]. For nearly a decade, PEDV outbreaks have occurred frequently around the world, inflicting significant damage on the pig industry.

Vaccination is currently the primary method of preventing and controlling PED epidemics. However, the emergence of more variant strains has resulted in a reduction in the classic vaccine’s efficacy. Hence, it is essential to explore novel approaches for averting and managing PEDV infection. In recent years, small-molecule inhibitors have become a popular research subject. Deehai conducted a screening of the FDA-approved small-molecule library of drugs to identify those that can suppress the activity of PEDV N protein [3]. Trichlorothiazide, D-biotin, and glutathione (GSH) were discovered to have considerable anti-PEDV potential. Furthermore, 2-deoxy-D-glucose (2-DG) is a glucose analogue that can hinder glycolysis by inhibiting glucose metabolism [4]. Additionally, it has been observed that 2-DG can disrupt viral protein translation during the primary phases of the PEDV lifecycle and the assembly of viral particles, leading to an impediment of the virus’s replication.

In late 2019, a novel coronavirus, known as Severe Acute Respiratory Syndrome Coronavirus 2 (SARS-CoV-2), emerged and quickly spread worldwide. The evolution of SARS-CoV-2 has been ongoing since its initial discovery in humans in December 2019, with an average rate of approximately two mutations per month observed in human isolates worldwide, which makes drug research targeting conservative protein sites extremely urgent [5,6]. Throughout the course of this pandemic, evidence confirmed that molnupiravir efficiently inhibits SARS-CoV-2 replication in human lung tissues [7]. Consequently, it became the first orally administered antiviral drug to receive approval for the treatment of mild-to-moderate SARS-CoV-2 infection in adult patients worldwide [8]. Molnupiravir, a biological prodrug of β-D-N(4)-hydroxycytidine (NHC), belongs to a kind of nucleoside analogue that exhibits a wide range of antiviral activities against various viruses, including SARS-CoV [9], SARS-CoV-2, Middle East respiratory syndrome coronavirus (MERS-CoV), influenza virus [10], respiratory syncytial virus (RSV) [11], and Ebola virus (EBOV) [12]. According to biochemical analyses, RdRp utilizes NHC triphosphate as the substrate instead of cytidine triphosphate or uridine triphosphate. The utilization of NHC during the RdRp synthesis of RNA based on synthesized templates can result in altered RNA products, since it directs the incorporation of G or A. [13]. This two-step mutagenesis mechanism may be applicable to multiple viral polymerases. At present, there are no published data on the effectiveness of this drug against PEDV.

In this study, our results demonstrate that molnupiravir effectively blocks PEDV replication in vitro and reverses the transcriptome changes caused by viral infection. These findings are expected to provide insights for the application of molnupiravir in anti-PEDV infection.

## 2. Material and Methods

### 2.1. Cells and Virus

Vero cells (ATCC CCL-81) and HEK-293T (ATCC CRL-3216) cells were cultured in Dulbecco’s Modified Eagle’s Medium (DMEM, Gibco, Grand Island, NE, USA); LLC-PK1 cells, provided by Shanghai Veterinary Research Institute, Chinese Academy of Agricultural Sciences, were cultured in Minimum Essential Medium (MEM, Gibco, USA). The media for all cell lines contained 10% fetal bovine serum (04-001-1A, BIOIND, Kibbutz Beit Haemek, Israel). The cells were kept in CO_2_ incubator at 37 °C. The PEDV strain used in this study was SHpd/2012 (GenBank accession nos.MN508818.1), and the Reed–Muench method was used to calculate the virus titers.

### 2.2. Compounds

Molnupiravir (CAS no. 2492423-29-5) was purchased from MedChemExpress, USA. Dimethyl sulfoxide (DMSO (60313ES60, YEASEN, Shanghai, China)) was used to dissolve the compound to obtain 20 mM stock solution. Coelenterazine-h was purchased from AAT Bioquest, (Pleasanton, CA, USA).

### 2.3. Antibodies and Plasmids

Primary antibodies included anti-PEDV N mouse mAb [14] (preserved in our laboratory), anti-Flag mouse mAb (M20008M, Abmart, Shanghai, China), anti-β-actin mouse mAb (T40104, Abmart, China). Secondary antibodies included Goat Anti-Mouse IgG-HRP (M21011, Abmart, China), Goat Anti-Mouse IgG AF 488 (M21011, Abmart, China).

The plasmids pcDNA4.0-Nsp7, pcDNA4.0-Nsp8, and pCAGGS-Nsp12, which express target protein containing a Flag tag, were provided by Shanghai Veterinary Research Institute, Chinese Academy of Agricultural Sciences. The pcDNA4.0-PRRSV-Nsp9 was constructed by our laboratory for this experiment. To obtain plasmid pPEDV-Gluc, which produces positive-strand of vRNA encoding Gaussia luciferase (Gluc), we adopted overlapping PCR to synthesize 5′UTR-Gluc-3′UTR. Subsequently, this sequence was inserted into pRetroX-tight-Pur vector at the BamHⅠ and EcoRⅠ sites. The primers for target-gene detection are listed in Table S1.

### 2.4. Cytotoxic Assay

The cytotoxicity of molnupiravir in cells was tested by CCK8 assay. The CCK8 cytotoxicity kit was purchased from TOPSCIENCE (Shanghai, China). Briefly, 96-well plates were used to culture cells until they reached 80% confluence after 24 h. Next, molnupiravir, diluted with cell-culture medium, was added by concentration from 1.5 μM to 384 μM. The blank control consisted of DMSO. After 24 h, CCK8 was added and incubated for 1 h at 37 °C of 5% CO_2_. Using the 722N visible spectrophotometer purchased from INESA (Shanghai, China), the absorbance at 450 nm was recorded.

### 2.5. Viral Life-Cycle Assays

#### 2.5.1. Anti-Entry Assays

In order to study the effect of molnupiravir on virus attachment, Vero cells in a 12-well cell-culture plate (2 × 10^5^ cells/well) were incubated with PEDV ([multiplicity of infection (MOI) = 0.1) and multiple concentrations of molnupiravir at 4 °C for 1 h to promote virus binding while preventing internalization. The blank control was prepared using DMSO. Next, the medium was discarded, and pre-cooled phosphate-buffered saline (PBS) was used to wash wells thrice. Virus infection levels were quantified by RT-qPCR.

In order to study the effect of molnupiravir on virus internalization, cells were infected in the same manner as described above. Next, the cells were treated with different concentrations of molnupiravir. The blank control was again prepared using DMSO. The samples were stored in CO_2_ incubator at 37 °C for 2 h. Subsequently, the media were discarded, and the cells were washed thrice. Virus infection was determined by RT-qPCR.

#### 2.5.2. Virus Replication Assays

To access viral replication, we seeded Vero cells into 6-well plates (2 × 10^6^ cells/well) for 24 h to obtain 90% cell density and washed them three times with PBS. Next, the cells were exposed to PEDV (MOI = 0.1) and incubated for 1 h at 37 °C and with 5% CO_2_ to ensure complete viral adsorption and penetration. The virus solution was discarded, and using PBS, plates were washed three times to ensure no virus remained in culture medium. Subsequently, we added different concentrations of molnupiravir to each sample while DMSO was prepared as the blank control. Finally, the samples were incubated in CO_2_ incubator at 37 °C until the control wells showed evident complete cytopathic effect (CPE). Characteristic morphological changes in Vero cells infected with PEDV were monitored and photographed. Furthermore, cells and supernatants were collected for viral load and titer assessment using RT-qPCR, indirect immunofluorescence, Western blotting, and the 50% tissue-culture infectious dose (TCID_50_) assay.

#### 2.5.3. Virus Release Assay

Vero cells were infected with PEDV (MOI = 0.1) for 1 h at 37 °C to ensure complete viral adsorption and penetration. Next, dishes with cultured cells were washed with PBS to eliminate any free virus. After infection for 10 h, the cells were then incubated with molnupiravir at concentrations of 24 μM, 48 μM, or 96 μM, or with DMSO as a control for a period of 2 h at 37 °C. Subsequently, the cell supernatant was collected to detect the mRNA copies of PEDV-N by RT-qPCR.

### 2.6. Indirect Immuno-Fluorescence Assay (IFA)

The cells were processed according to method 2.5. The cells were washed thoroughly three times with PBS. Next, the cells were fixed using 80% cold ethanol for a period of 1 h at 4 °C. Fixative solution was aspirated, cells were washed three times, and mouse anti-PEDV N mAb was added. The mixture was incubated at 37 °C for 1 h. The samples were washed with PBS thrice again, and Goat Anti-Mouse IgG AF 488 was added to plates. Mixture was incubated at 37 °C for 45 min and protected from light. The Invitrogen EVOS FL Auto Cell Imaging System was used to obtain images after thoroughly washing the samples with PBS.

### 2.7. Western Blots

To collect and lyse cells, radio immunoprecipitation assay (RIPA) lysis buffer containing 1 mM phenylmethanesulfonyl fluoride (PMSF) was used for a time period of 30 min at 4 °C. Next, sodium dodecyl sulfate-polyacrylamide (SDS) loading buffer was added to the cell lysates and boiled for 10 min. The protein samples were then separated using 12% alkyl sulfate and 10% polyacrylamide gel electrophoresis before they were transferred onto polyvinylidene difluoride (PVDF) membranes. Next, the membranes with transferred protein were blocked using tris-buffered saline with Tween 20 (TBST) and 5% skimmed milk for a period of 2 h. The membranes were then incubated with mouse anti-PEDV N mAb, mouse anti-β-actin mAb, or mouse anti-Flag mAb at a low temperature of 4 °C for a duration of 12 h. Following five washes with TBST, the membranes were incubated with Goat Anti-Mouse IgG-HRP for a period of 45 min. Next, the membranes were washed three times with TBST and the signal was detected using the ECL reagent (SQ201, Shanghai Epizyme Biomedical Technology, Shanghai, China). Finally, the protein staining on PVDF membranes was analyzed by the Tanon-5200 multi infrared imaging system.

### 2.8. RT-qPCR

To extract total RNA from the cells, we used the EZ-10 DNAaway RNA Mini-Preps Kit (no. B618133, Sangon Biotech, Shanghai, China) and performed reverse transcription using the Hifair^®^ Ⅱ 1st Strand cDNA Synthesis SuperMix (11120ES60, YEASEN, Shanghai, China) following the manufacturer’s instructions. For qPCR, we utilized Hieff^®^ qPCR SYBR Green Master Mix (11201ES03, YEASEN, China) and performed the analysis on a CFX96 Real-Time System (Bio-Rad). The sequence for qPCR primers can be found in Table S1. To determine the relative expression of threshold cycle (CT) value, we analyzed the qPCR results using the 2^−ΔΔCt^ method.

### 2.9. TCID_50_ Assay

In order to assess the virus titers, we inoculated Vero cells on a 96-well plate and allowed them to grow to a monolayer. Next, the cells were rinsed with PBS for three repetitions before they were exposed to PEDV virus solution, which had been serially diluted by a factor of 10, with a total of eight replicates. Subsequently, the plates were kept at 37 °C with 5% CO_2_ for five days and monitored on a daily basis. The wells were marked as PEDV-positive once CPE appeared. The PEDV titration was calculated by TCID_50_ following the Reed–Muench method.

### 2.10. Gluc Activity Assay

The Coelenterazine-h we purchased was in powder form. To prepare the Coelenterazine-h solution, we dissolved it in propylene glycol until the concentration reached 5 mM as a stock solution. Before each assay, the stock solution was diluted in PBS to a concentration of 50 μM and incubated in the dark for 30 min at room temperature. For the luminescence test, we added 10 μL of supernatant to each well of a 96-well plate, which was white and opaque. Next, we injected 60 μL of working fluid and measured luminescence for 0.5 s using BioTek Synergy2 multifunctional microplate readers.

### 2.11. RNA-Seq Analysis

To generate sequencing libraries, total RNA was extracted using Invitrogen Life Technologies’ Trizol reagent and Thermo Scientific’s NanoDrop spectrophotometer was used to assess its concentration, purity, and integrity. The library-generation process involved purifying mRNA from the total RNA using Poly-T oligo-attached magnetic beads, followed by fragmenting it in an Illumina proprietary fragmentation buffer containing divalent cations at a high temperature. Subsequently, we used SuperScript II and random oligos to synthesize first-strand cDNA. The DNA Polymerase I and Rnase H were utilized for the second-strand cDNA synthesis. Exonuclease/polymerase activities were used to convert the remaining overhangs into blunt ends, and the enzymes were removed. Adenylated Illumina PE adapter oligos were ligated with the 3′ ends of DNA fragments for hybridization. The libraries were purified using Beckman Coulter’s AMPure XP system to acquire cDNA fragments with lengths of 400–500 bp. The DNA fragments with adapter molecules on both ends were selectively enriched in a 15-cycle PCR reaction by using Illumina PCR Primer Cocktail. Subsequently, the products were purified again using the AMPure XP system, and the Agilent high-sensitivity DNA assay on a Bioanalyzer 2100 system was utilized to quantify them before performing sequencing on Illumina’s NovaSeq 6000 platform. Finally, further analyses, such as expression differences, enrichment, and clustering, were conducted on the samples.

### 2.12. Statistical Analysis

All data were presented as the mean ± SD from at least three independent experiments and were analyzed using GraphPad Prism 8.0.1 software. The two configurations were deemed to have statistical significance if *p* < 0.05 (*), *p* < 0.01 (**), *p* < 0.001 (***) and *p* < 0.0001 (****). The “N.s” is used to indicate lack of statistical significance.

## 3. Results

### 3.1. Significant Inhibitory Effect of Molnupiravir on PEDV Proliferation

**Figure 1:**
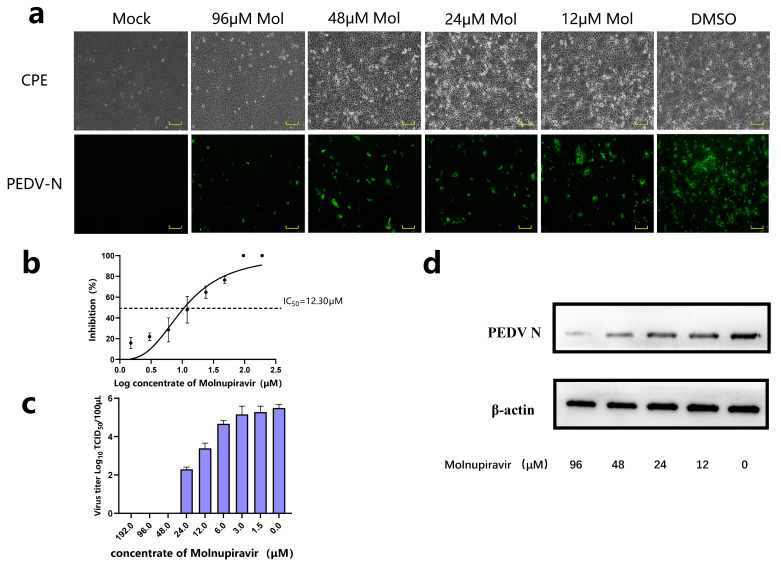
Effects of molnupiravir on PEDV proliferation. Cells from the Vero lineage were subjected to PEDV virus at an MOI of 0.1 and incubated for 1 h at 37 °C. Next, varying concentrations of molnupiravir were introduced. (**a**) Antiviral activity of molnupiravir against PEDV was assessed through CPE observation and IFA. Mol, molnupiravir (scale bar: 100 μm). (**b**) Dose–response analysis of molnupiravir in Vero cells. Inhibition rates were calculated by relative quantification of PEDV N protein mRNA. (**c**) Following the administration of molnupiravir, the titers of virus present in the supernatant exhibited a noticeable decrease, which was contingent upon the dose used. (**d**) Western blot identification in Vero cells. The PEDV-N-protein expression was influenced by molnupiravir. Mol, molnupiravir.

We first evaluated the cytotoxicity of molnupiravir based on the CCK8 assay. As shown in Figure S1, this compound is generally safe to apply to Vero cells, HEK-293t cells, and PK1 cells. Next, we started to investigate its effect on PEDV proliferation. The Vero cells were exposed to PEDV at 37 °C for 1 h and treated with varying concentrations of molnupiravir. Initially, the antiviral effects of the drugs were determined based on the cell morphology. As shown in Figure 1a, the cells treated with high drug concentrations retained normal morphologies, similar to those of the mock cells. In contrast, with decreasing drug concentrations, more CPE, including swelling, rounding, shrinking, and syncytia formation, emerged. This observation leads to the conclusion that the addition of molnupiravir can effectively preclude the CPE induced by PEDV. The IFA results corroborate these findings, in that the fluorescence intensity in the well without the molnupiravir was significantly higher, and notably reduced after treatment with the drug.

This study employed RT-qPCR and TCID50 assays to respectively quantify the viral loads and titers in varying experimental groups. The molnupiravir exhibited a dose-dependent decrease in both the viral genomic copies and the viral titer, (Figure 1b,c). Furthermore, the half-maximal inhibitory concentration (IC_50_) value was 12.30 μM. It is noteworthy that the Western blot analysis led to congruent findings, wherein the concentration-dependent inhibition of the PEDV-N-protein expression by the molnupiravir was found to be obvious compared to that in the control group (Figure 1d). The PK1 cells were utilized to investigate the efficacy of the molnupiravir on pig cells, with experiments similar to those performed previously. Figure S2 indicates that molnupiravir similarly exhibited anti-PEDV properties in the PK1 cells. Despite the drug concentration reaching 160 μM, its inhibitory efficiency was still insufficient, at less than 80% (Figure S2b), which indicated that its efficacy in the PK1 cells was less impressive than in the Vero cells.

In summary, the aforementioned findings suggest that molnupiravir possesses the ability to hinder the viral replication, protein expression, and infectivity of virus particles.

### 3.2. Significant Antiviral Effect of Molnupiravir Only on PEDV Replication Stage

**Figure 2:**
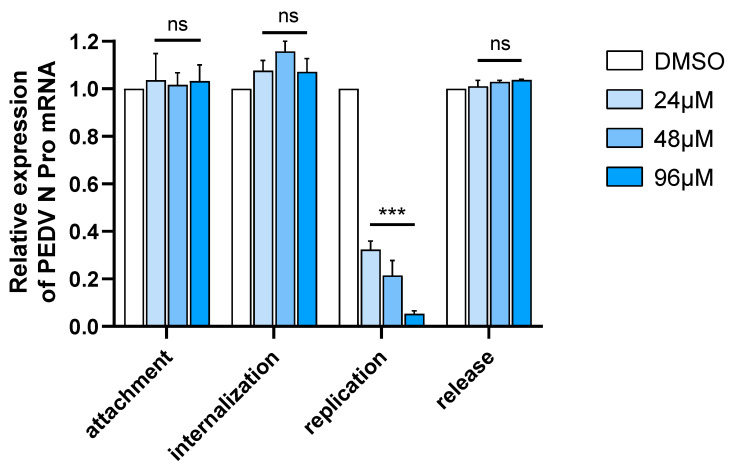
Influence of molnupiravir on PEDV attachment, internalization, replication, and release. Synthesized PEDV N Pro mRNA levels were assessed by RT-qPCR. Asterisk (*) indicates a significant difference among them (*** *p*  <  0.001). The “ns” is used to indicate lack of statistical significance.

Antiviral drugs commonly function by intervening in virus adsorption, internalization, replication, and release processes. The study compared the relative expression levels of N and GAPDH between the treatment and control groups to evaluate molnupiravir’s effects. The RT-qPCR results indicated that the expression of N mRNA was inhibited only when the drug was administered during the replication phase (Figure 2). In other words, the drug had no significant effect on the attachment, internalization, or release of PEDV, at all the tested concentrations.

### 3.3. PEDV-RdRp-Gluc Reporter System and Molecular Modeling to Confirm That Molnupiravir Acts on PEDV RdRp

**Figure 3:**
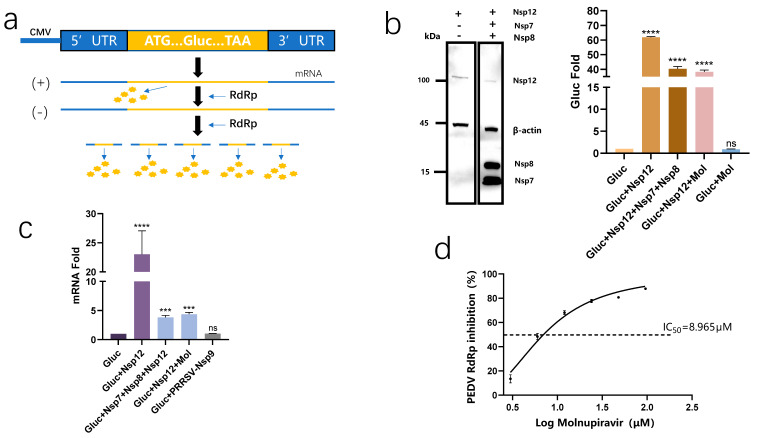
PEDV RdRp activity was inhibited by molnupiravir. (**a**) Schematic diagram of PEDV−RdRp−Gluc reporter system. The sense strand of the Gluc−expression cassette was utilized to bridge the PEDV 5′UTR and 3′UTR. When this sequence was transcribed to mRNA, RdRp recognized it and started to synthesize more Gluc RNA. Next, the replicated plus−sense Gluc RNA was translated to express Gluc. (**b**) The 293T cells were co-transfected with plasmids. (Gluc:Nsp12:Nsp7:Nsp8 = 1:10:30:30). Expression of the PEDV Nsp12, Nsp7 and Nsp8 gene with Flag was detected with an anti-Flag antibody by Western blot 24 h after transfection. The β−actin was identified as the internal control for loading normalization. The supernatants were used in Gluc activity assay. (**c**) The quantification of Gluc RNA levels was conducted through RT-PCR analysis on the HEK−293T cells that were transfected with the indicated plasmid. (**d**) Molnupiravir inhibited PEDV-Gluc in a dose-dependent manner. The HEK−293T cells were transfected with PEDV−Gluc and Nsp12 plasmid at a ratio of 1:10. Six hours post−transfection, cells were cultured with serially diluted molnupiravir. These outcomes represent the mean of three independent experiments. Gluc, Gaussia luciferase. Mol, molnupiravir. Asterisk (*) indicates a significant difference among them (*** *p*  <  0.001; **** *p*  <  0.0001). The “ns” is used to indicate lack of statistical significance.

We developed a cell-based PEDV-RdRp-Gluc reporter system to evaluate the impact of molnupiravir on PEDV RdRp. The reporter vector was created by building a Gluc gene placed between the 5′ and 3′ untranslated regions (UTRs) of the PEDV, as illustrated in Figure 3a. The RdRp has a certain template specificity, which is mainly manifested in the UTR sequence of the template and the secondary structure it forms [15]. Gluc transcription is initiated by the cytomegalovirus (CMV) promoter, which increases Gluc-mRNA and Gluc-protein levels through RdRp expression. The amplified Gluc activity on the luminescence test suggested PEDV RdRp activity. Following the transfection of the reporter vector and pCAGGS-Nsp12, there was a 60-fold elevation in Gluc activity within the cells compared to the cells transfected with the control reporter vector. The reporter-plasmid-to-RdRp-vector ratio was maintained at 1:10 to ensure low background values. As reported previously, SARS-CoV-2’s Nsp7 and Nsp8 are subunits of the RdRp holoenzyme. Consequently, Nsp7 and Nsp8 were also transfected into the PEDV-Gluc-reporter-expressing cells with Nsp12. Surprisingly, the presence of Nsp7 and Nsp8 led to an increase in Gluc activity, albeit lower than that of the PEDV-Gluc-reporter-expressing cells transfected with Nsp12 alone. The increase in Gluc activity in the presence of RdRp was also supported by the RT-qPCR results (Figure 3c). The PEDV-RdRp-Gluc reporter system without Nsp7 or Nsp8 was selected as a result. In order to verify the specificity of the system aimed at the detection of PEDV, the Nsp9 protein sourced from the porcine reproductive and respiratory syndrome virus (PRRSV) was employed as a negative control. The Nsp9 protein possesses RdRp enzyme activity but is not related to PEDV [16]. The Nsp9 protein of the PRRSV did not increase the Gluc translation level (Figure 3c and Figure S3). Based on these data, we reached the conclusion that the PEDV RdRp was able to enhance the Gluc activity.

In order to confirm that the molnupiravir targeted the PEDV RdRp to inhibit viral replication, the PEDV-RdRp-Gluc reporter system was used. During the pre-experiments, the interference of background values in the presence of the drug was eliminated (Figure 3b). The plasmids (Gluc, Nsp12) were co-transfected into the cells and different concentrations of molnupiravir were added into the medium at 6 h post-transfection. These findings demonstrate that the molnupiravir inhibited the Gluc activity levels in a dose-dependent manner (Figure 3d), suggesting the potential for decreasing PEDV RdRp activity with the drug.

Additionally, we uploaded the Nsp12 sequence of the PEDV shpd/2012 strain to the AlphaFold2 online website for protein-structure prediction. Subsequently, we investigated the binding mode of molnupiravir’s active form, EIDD-1931, using AutoDock Vina v1.2.0 software. Furthermore, PoseView was utilized to obtain a graphical two-dimensional representation of the interaction between the PEDV Nsp12 and the EIDD-1931. According to the docking results, the EIDD-1931 formed a cation-π interaction with the 574Arg, and four hydrogen bonds with 615Asp, 674Ser, and 752Asp (Figure S4). These findings imply that molnupiravir can affect RdRp activity by targeting the protein directly.

### 3.4. Molnupiravir Increases Mutation Rates in PEDV gRNA

**Figure 4:**
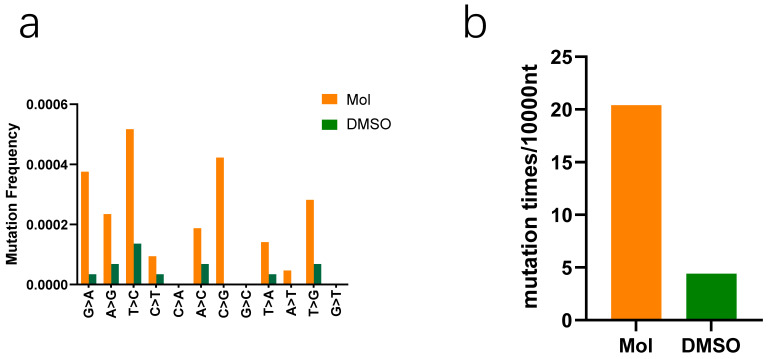
Molnupiravir increases mutation rates in PEDV gRNA. (**a**) The occurrence rate of all mutation types in both groups. (**b**) Mutation rates were calculated by sequencing results. Mol, molnupiravir.

According to the two-step mutagenesis mechanism, once converted to its active form within the cellular environment, molnupiravir inserts itself into the viral RNA chain, which leads to the production of a large number of mismatched chains when used as a template, which causes catastrophic errors during viral replication [17]. Therefore, we evaluated the effect of the molnupiravir on the mutation rates of the PEDV gRNA. The Vero cells were infected with PEDV and either incubated with 24 μM of molnupiravir or not. When the CPE appeared in the control group, the gRNA was collected and we randomly selected 725-nucleotide (nt) fragments of PEDV Nsp12 sequence from both groups to amplify. The fragments were cloned into a vector. Next, the monoclonal colonies of 50 fragments were randomly chosen and sequenced. In a manner that was consistent with other reports, the molnupiravir increased the frequency of mutations in this fragment (Figure 4). We identified 74 mutations (20.4 mutations per 10,000 nt) in the fragments from the drug-treated group, and only 16 mutations (4.4 mutations per 10,000 nt) appeared in the fragments from the mock-treated group. The mutation rates were increased by approximately 4.6 times by the molnupiravir. However, this ratio was probably even higher, since equal numbers of mutations were probably derived from the reverse transcription and PCR. In summary, the molnupiravir induced higher mutation rates in the PEDV gRNA.

### 3.5. Transcriptional Analysis after Molnupiravir Treatment

**Figure 5:**
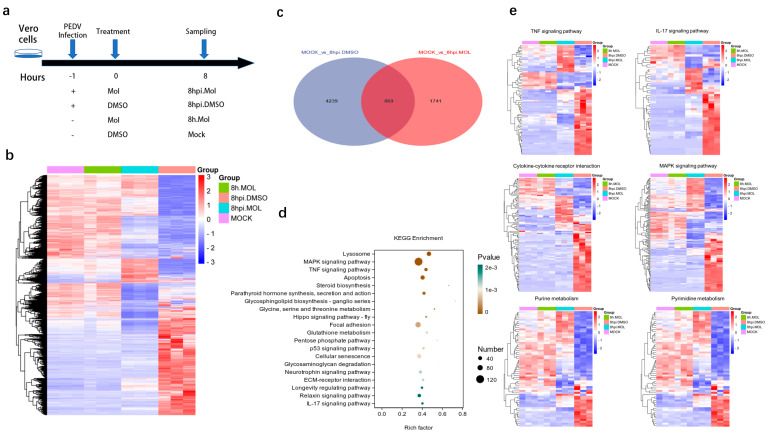
Transcriptional analysis of molnupiravir treatment. (**a**) Transcriptomic−experimental-treatment schematic diagram (MOI = 0.2). Mol, molnupiravir. (**b**) Cluster−analysis heatmap of transcription levels across all samples. The significantly and differentially expressed genes (differential expression fold | log2FoldChange | > 1, significant *p*-value < 0.05) between infection and mock are shown. Conditions include 8 h after infection for Vero cells with or without molnupiravir treatment. Genes were clustered using the complete method. (**c**) Common differential genes between mock versus 8hpi.DMSO and mock versus 8hpi.MOL groups. (**d**) Differential genes from groups “8hpi.DMSO vs. 8hpi.Mol” KEGG−enrichment scatter plot. (**e**) Heat map of the genes enriched in TNF signaling, IL-17 signaling, cytokine−cytokine receptor interaction, MAPK signaling, purine metabolism or pyrimidine metabolism.

The RNA-seq can analyze genetic information for all transcripts in a biological niche. We used this technology to access the transcriptome changes in the Vero cells to deeply explore the mechanism through which molnupiravir inhibits PEDV infection. Vero cells infected with or without PEDV and treated with molnupiravir or DMSO for 8 h were used for the transcriptional analysis (Figure 5a). The transcriptome data were uploaded to NCBI, with the sequence number SRP435862. A cluster analysis of the differential genes showed that the molnupiravir treatment generally shifted the transcriptome back to the mock-treatment control group (Figure 5b). The PEDV infection altered the RNA levels of 5108 genes, and 83% of these genes returned to normal levels after the molnupiravir treatment (Figure 5c). This indicated that the molnupiravir treatment eliminated most of the transcriptome changes induced by the PEDV infection. Focusing on the treatment with molnupiravir for 8 h after infection, we observed that the four pathways with the most significant enrichment of differential genes were subordinate to innate immunity, including the MAPK and TNF pathways, and the host’s antiviral mechanisms, such as lysosome and apoptosis (Figure 5d). These results indicate that molnupiravir can regulate not only the innate immune pathway, but also the lysosome and apoptosis pathways, to achieve antiviral effects. Furthermore, the molnupiravir treatment was found to frequently reverse the effects of the PEDV infection in genes belonging to key metabolic pathways in the enrichment analysis (Figure 5e).

## 4. Discussion

The spread of PEDV has significantly hindered the global pig-breeding industry. As the effectiveness of traditional vaccines has proven to be insufficient against new mutant strains, the demand for effective therapeutic drugs has become pressing. Previous studies showed that molnupiravir is a crucial drug in combatting the SARS-CoV-2 pandemic. Its antiviral function targets RdRp, giving it inhibitory potential against a range of viruses. Our research confirmed that molnupiravir is capable of hindering PEDV infection in vitro.

The course of coronavirus infection encompasses attachment, internalization, replication, and release. We evaluated the effects of molnupiravir on these stages through RT-qPCR, Western blot, and IFA. Our findings indicated that molnupiravir only affects replication and reduces the production of PEDV genes and proteins in a dose-dependent manner, with an IC_50_ of 12.30 μM. Intriguingly, the RT-qPCR detected PEDV mRNA in the presence of 48 μM of molnupiravir, but not of supernatant titers. We speculated that molnupiravir’s incorporation into the viral genome leads to a high number of mutations, resulting in the loss of viral infectivity. Additionally, we identified mutations in a random sequence of PEDV, regardless of whether it was treated with molnupiravir. The experimental results supported our speculation that molnupiravir increases the mutation rates in PEDV gRNA. Previous studies reported that molnupiravir’s active substance can induce high levels of mutations in the genome of the Venezuelan equine encephalitis virus (VEEV) [18]. Similarly, a clinical genomic analysis study of AGILEIIa published in *Nature Communications* reported a significant increase in the mutation rate of SARS-CoV-2, especially in the mutations between U and C, due to the use of molnupiravir [19]. These results align with our findings. We conducted similar experiments on the PK1 cell line and found that molnupiravir exhibited only partial antiviral activity. We suspect that molnupiravir may have insufficient ability to convert into active substances in this cell line.

Zhao et al. established a cell-based assay to discover inhibitors of SARS-CoV-2 RdRp [20]. We constructed a similar system to investigate the impact of molnupiravir on PEDV RdRp activity. During the construction of this system, we found that Nsp7 and Nsp8 showed no significant effect on the activity of PEDV RdRp in the system, which was different from the SARS-CoV-2 system. This alteration was induced by either a different mechanism of the coronavirus RdRp or the use of a different experimental methods for establishing the reporting system. We used this system to test the molnupiravir and found it also inhibited Gluc activity in a dose-dependent manner, with an IC_50_ = 8.965 μM, which was close to the IC_50_ value against PEDV infection in the Vero cells. Our system holds promise for identifying potential inhibitors of PEDV RdRp.

As a mature technology, RNA-seq is often used in the study of PEDV–host interaction. In this experiment, the heat map of the differential genes showed that the molnupiravir recovered the transcriptome changes caused by the PEDV infection. The damage to the host caused by coronavirus is usually manifested by an excessive inflammatory response [21]. We observed that the transcriptional profiles of inflammation-related metabolic pathways were changed by the PEDV infection, in addition to the recovery due to the molnupiravir. Generally, viruses hijack the host’s nucleotide metabolism for their own replication [22]. Molnupiravir treatment also moderates the transcriptional profile changes in nucleotide-related metabolic pathways. It was reported that β-coronavirus can convert the lysosomes of host cells into containers for virus storage and release [23]. Therefore, newly synthesized β-coronavirus particles are not released through the classical secretory pathway, but are transported to lysosomes and accumulate there until they are released in large quantities by lysosomal exocytosis. We observed that the differential genes were most significantly enriched into the lysosome pathway, which led us to suspect that the molnupiravir regulated the interaction between the PEDV and the host lysosomes to protect the host, although PEDV belongs to α-coronavirus.

Although we demonstrated the effective inhibition of molnupiravir against PEDV in Vero cells, its potency is less efficient in PK1 cells, so experiments on piglets are needed to ensure its reliability in the future. As the first oral COVID-19 drug globally, molnupiravir is expensive for a single treatment, and cost factors may prevent it from becoming the preferred drug for treating PED at this moment. However, the key to promoting this drug in the breeding industry is to constantly optimize its synthesis process and reduce costs. We hope that this goal can be achieved in the future.

## 5. Conclusions

In conclusion, we found that molnupiravir has anti-PEDV activity by inhibiting RdRp activity and that it can recover changes in host global transcription profiles in response to PEDV infection. This study shows that molnupiravir is a potential anti-PEDV drug.

## Data Availability

The transcriptome data were uploaded to NCBI, with the sequence number SRP435862.

## Supplementary Materials

The following supporting information can be downloaded at: https://www.mdpi.com/article/10.3390/v15061317/s1, Figure S1: Cytotoxicity of molnupiravir on Vero cells, HEK-293t cells and PK1 cells; Figure S2: The antiviral effect of molnupiravir on PK1 cells; Figure S3: PRRSV Nsp9 protein eukaryotic expression; Figure S4: Docking of EIDD-1931 with PEDV. Table S1: List of primers used in this study.
