# Supplementary material for: Molnupiravir Inhibits Porcine Epidemic Diarrhea Virus Infection In Vitro
Source: Viruses. 2023 Jun 2;15(6):1317. doi: 10.3390/v15061317 (PMC10302869; doi:10.3390/v15061317)
Supplement: Supplementary file 1 [file viruses-15-01317-s001.zip › viruses-2426779-supplementary.pdf]

Supplementary Materials:

Table S1. List of primers used in this study.

Primer Name	Sequence (5′-3′)
PEDV-5′UTR-F	CCTGGAGAAGGATCCACTTAAAAAGATTTTCTATCTAC
PEDV-5′UTR-R	AACTTTGACTCCCATAGCCGACAGTTACTGGTTTCAAA
Gluc-F	ATGGGAGTCAAAGTTCTGTTTGC
Gluc-R	TTAGTCACCACCGGCCCCCTTGA
PEDV-3′UTR-F	GCCGGTGGTGACTAAACAATGTTTGACTGGCTTGTCCT
PEDV-3′UTR-R	AGCCGGCGCGGCCGCGTGTATCCATATCAACACCGTCA
PEDV-N-qPCR-F	CGCAAAGACTGAACCCACTAAC
PEDV-N-qPCR-R	TTGCCTCTGTTGTTACTTGGAGAT
qPCR-GAPDH-F-monkey	ACAACAGCCTCAAGATCGTCAGC
qPCR-GAPDH-R-monkey	GTGGCAGTGATGGCGTGGAC
qPCR-Gluc-F	CACGCCCAAGATGAAGAAGT
qPCR-Gluc-R	GAACCCAGGAATCTCAGGAATG
qPCR-293t-GAPDH-F	TGGGTGTGAACCATGAGAAG
qPCR-293t-GAPDH-R	GTCCTTCCACGATACCAAAGT
mutation-F	GGTACCGAGCTCGGATCCTGAACTGTGTTCGCCTGAAGAA
mutation-R	GCTCCACCGCCTCCCTCGAGTTCCAAACTATACCTAACTG

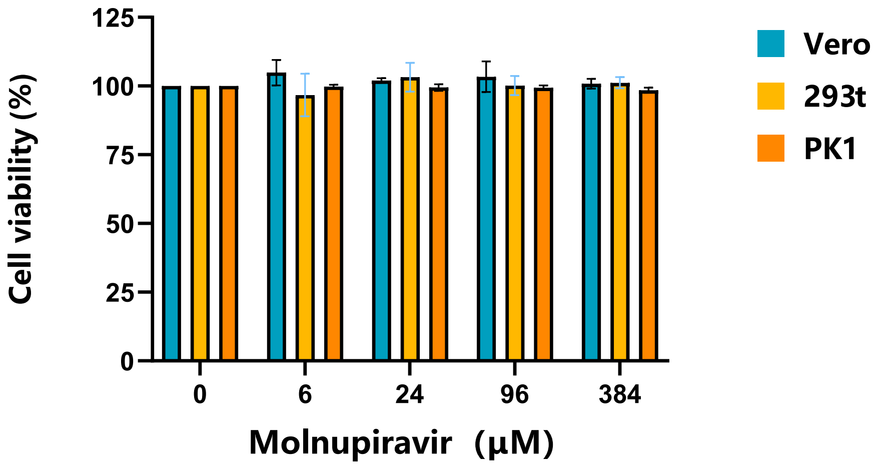

Figure S1. Cytotoxicity of molnupiravir on Vero cells, HEK-293t cells and PK1 cells. Cytotoxicity of molnupiravir toward Vero cells, 293t cells and PK1 cells was assessed by CCK8 assay. The data from cells cultured without molnupiravir was set to 100%. Each date represents the average of three biological replicates.

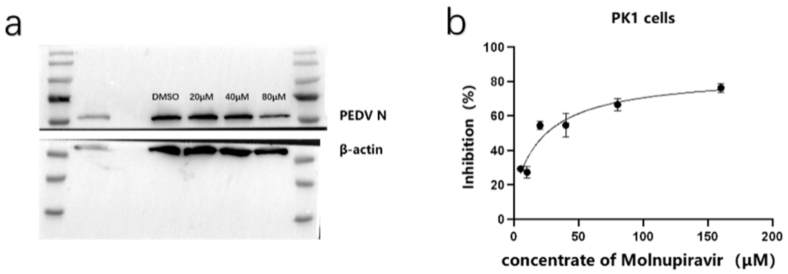

Figure S2. The antiviral effect of molnupiravir on PK1 cells (a) Western blot identification in PK1 cells. PEDV N protein expression was influenced by molnupiravir.(b) Dose–response analysis of molnupiravir in Vero cells.

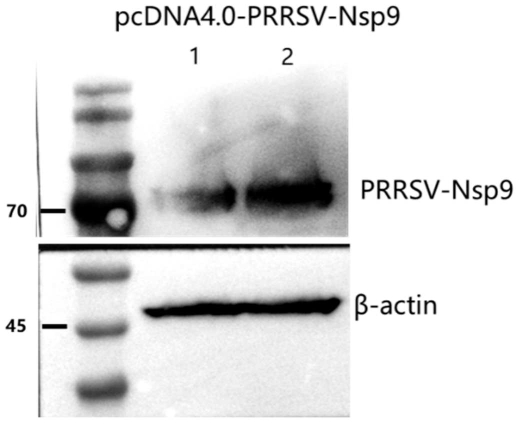

Figure S3. PRRSV Nsp9 protein eukaryotic expression.

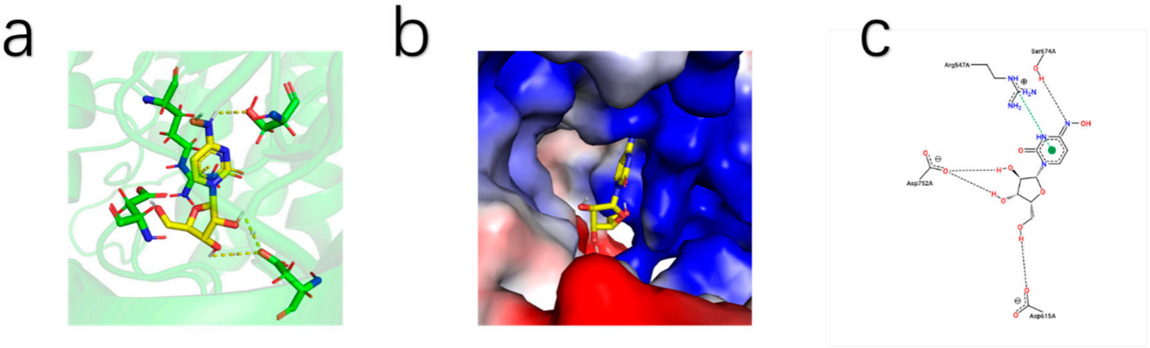

Figure S4. Docking of EIDD-1931 with PEDV (a) Proposed binding mode of EIDD-1931 against PEDV Nsp12. (b) Electrostatic potential map of PEDV Nsp12 with EIDD-1931. (c) Interaction between the PEDV Nsp12 and EIDD-2801 by PoseView. The corresponding diagram produced by the website version of PoseView (https://proteins.plus/).
